# Zinc and linoleic acid pre-treatment attenuates biochemical and histological changes in the midbrain of rats with rotenone-induced Parkinsonism

**DOI:** 10.1186/s12868-018-0429-9

**Published:** 2018-05-09

**Authors:** Ngala Elvis Mbiydzenyuy, Herbert Izo Ninsiima, Miriela Betancourt Valladares, Constant Anatole Pieme

**Affiliations:** 1 Department of Physiology, Faculty of Biomedical Sciences, Kampala International University, Western Campus, Ishaka, Uganda; 2 Department of Human Physiology, University of Medical Sciences, Camagüey, Cuba; 3 Department of Biochemistry and Physiological Sciences, Faculty of Medicine and Biomedical Sciences, University of Yaounde I, Yaounde, Cameroon

**Keywords:** Neuroprotection, Antioxidant, Brain, Nutrition, Ageing

## Abstract

**Background:**

Studies have suggested the supplementation of Zinc and Linoleic acid in the management of neurodegenerative disorders but none has investigated the combined effects. Little is known about the neuroprotective effects of either Zinc or Linoleic acid or their combination against development of Parkinsonism. This study was designed to investigate the neuroprotective effects of Zinc and Linoleic acid in rotenone-induced Parkinsonism in rats.

**Methods:**

Thirty-six young adult female rats weighing 100–150 g divided into six groups were used. Rats were induced with Parkinsonism by subcutaneous administration of rotenone (2.5 mg/kg) once a day for seven consecutive days. The rats received dimethyl sulfoxide (DMSO)/Olive oil or rotenone dissolved in DMSO/Olive oil. Groups III and IV received Zinc (30 mg/kg) or Linoleic acid (150 µl/kg) while group V received a combination of both, 2 weeks prior to rotenone injection. Groups II and VI served as negative (rotenone group) and positive (Levodopa groups) controls respectively. Oxidative stress levels were assessed by estimating Lipid peroxidation (MDA), total antioxidant capacity, Superoxide dismutase, reduced Glutathione (GSH), glutathione peroxidase and catalase in the midbrain. Histological examination was done to assess structural changes in the midbrain.

**Results:**

There was a significant prevention in lipid peroxidation and decrease in the antioxidant status in intervention-treated groups as compared to the rotenone treated group. In addition, histological examination revealed that Parkinsonian rat brains exhibited neuronal damage. Cell death and reduction in neuron size induced by rotenone was prevented by treatment with zinc, linoleic acid and their combination.

**Conclusion:**

These results suggest that zinc and linoleic acid and their combination showed significant neuroprotective activity most likely due to the antioxidant effect.

## Background

Parkinsonism is a general term that refers to neurological disorder that causes movement disorders. These disorders include supranuclear palsy, vascular Parkinsonism, dementia with Lewy bodies, corticobasal degeneration and drug induced-Parkinsonism [44]. The pathophysiology of the latter is related to drug-induced changes in the basal ganglia motor circuit secondary to dopaminergic neuron destruction [21]. The consequence of nigro-striatal fibre degeneration is a loss of nerve endings in the striatum which contain tyrosine hydroxylase, and thus a reduced production of dopamine in the striatum. This produces tremor bradykinesia, rigidity and postural instability [18].

The aetiology of PD is considered to be associated with environmental exposures to various factors and genetic mutations [10]. Suggested environmental factors related to the aetiology of PD include exposure to herbicides and pesticides, intake of contaminated well-water and neurotoxins for example rotenone. Oxidative stress has been suggested as the common underlying mechanism in both sporadic and genetic cases of PD. This leads to cellular dysfunction [19]. The oxidative stress brings about death of dopaminergic neurons in the substantia nigra pars compacta. The initiators of these cascade of processes associated with generation of oxidative stress in the nigral dopaminergic neurons are thought to be the reactive oxygen species (ROS) produced during inflammation of the neurons, dysfunction of the mitochondria and metabolism of dopamine [22]. Micronutrients and trace elements have been described as key components in the combat against these ROS and hence the onset and progression of neurodegenerative disorders [5].

Current treatment of Parkinson’s disease (PD) is based on dopamine replacement therapy, but chronic administration may cause motor fluctuations and dyskinesias, increased free radical formation, accelerating neuronal degeneration in some PD patients. Studies in rodents showed that levodopa/carbidopa do not offer neuroprotection as well. Several agents have been investigated for neuroprotection against development of Parkinson disease or for slowing down the degeneration dopaminergic neurons, yet none has successfully offered neuroprotection.

Zinc homeostasis has been implicated in several processes related to brain aging and the onset and development of age-related neurodegenerative disorders. Serum Zinc did not significantly correlate with age of onset and duration of the disease in a study of Zinc status in PD patients [56] suggesting that Zinc does not play any role. Studies have found that Zinc deficiency accompanies many cases of PD as shown by significant low levels of Zinc in the cerebrospinal fluid (CSF) of PD patients. Zinc has been shown to play an important role in protecting dopaminergic neurons against free radicals and toxins from the environment by stimulating metallothionein production [26]. Another micronutrient essential for influencing signal transduction, neurochemistry, enzymes, membrane proteins and gene expression is the omega-6 polyunsaturated fatty acids of which Linoleic acid is the major long chain PUFA [7]. Omega-6 PUFAs also modulates transmission in cholinergic, serotoninergic and dopaminergic systems. Dopamine storage vesicle formation have been shown to significantly reduce in prolonged omega-6 PUFA deficiency [58]. Omega-6 PUFA is involved in inflammation, neurotrophic support, and oxidative stress through modulation of expression of genes responsible for that. Despite all these, the neuroprotective effects of omega-6 PUFA in PD are yet to be investigated elaborately [30].

There is therefore the need to investigate the potentials of micronutrients and trace elements offering protective effects in the brain or slowing down the degeneration processes that occur in toxin-induced neurodegeneration.

Using the hypothesis that mitochondrial dysfunction and oxidative injury underlie neurodegeneration in PD; the inclusion of metabolic modifiers may provide an alternative and early intervention approach. In order to investigate this we used a toxin-induced model of Parkinsonism to assess the protective effects of the trace element Zinc and micronutrient Linoleic acid. These agents may provide a potential neuroprotective therapy aimed for use as a prophylaxis to delay the onset or halt the progressive nature of Parkinsonism.

## Methods

### Chemicals and drugs

Rotenone (Sigma-Aldrich, St Louis, SA) was dissolved in 1:1 (v/v) dimethylsulfoxide (DMSO, Sigma-Aldrich, St Louis, SA). Zinc dust (10 g) (Sigma-Aldrich, St Louis, SA) was dissolved in distilled water; Linoleic acid (Sigma-Aldrich, St Louis, SA). All other chemicals and reagents were obtained from the Biochemistry and Physiology laboratories of the University of Yaounde 1 Cameroon and were of analytical grade.

### Animals

Female wistar rats were used in the present study. Their weight ranged between 100 and 150 g. Rats were housed in groups of six in stainless steel cages under hygienic laboratory conditions (temperature of 25 °C and reversed 12/12 h light/dark cycle). Water and food pellets were given ad libitum. All the experimental protocols were approved by the Institutional Animal Care and Ethics Committees at the Kampala International University Western Campus and Faculty of Medicine and Biomedical Sciences, University of Yaounde 1, Cameroon under the reference number No 2017/01/699/CE/CNERSH/SP.

### Experimental design

Thirty-six young adult female rats aged 8–12 weeks and weighing 100–150 g, obtained from the animal house, Department of Physiological Sciences and Biochemistry University of Yaounde I, Cameroon and acclimated in a room at temperature of 25 ± 1 °C were used. Rotenone (2.5 mg/kg dose, s.c.) was employed to induce experimental Parkinsonism [52]. Rotenone was prepared to be injected in a volume of 1 ml/kg body weight. The rotenone solution was first prepared as a 50X stock in 100% dimethylsulfoxide (DMSO) by dissolving 125 mg of rotenone in 1 ml of DMSO. 40 µl of the stock solution was then diluted in 1960 µl of olive oil. The solution was vortexed to create an emulsion of the DMSO containing rotenone and Olive oil. Fresh solution was prepared every 2–3 times in a week. Rotenone is sensitive to light, so it was stored in small vials and protected from light by wrapping the vials with foil papers and kept in refrigerator. Before administering the Rotenone solution to rats, the vials were inverted several times or vortexed to obtain a uniform mixture with the DMSO/Olive oil. Each rat received a volume of 1 ml/kg of Rotenone and control animals received the vehicle only (Olive oil/DMSO).

Group I (normal control) received the vehicle Olive oil/DMSO once a day through the study period of 3 weeks; Group II (negative control) as described by Fujikawa et al. [13] and Ojha et al. [33] received 2.5 mg/kg of rotenone subcutaneously once a day consecutively for the last 7 days of the experiment. Group III received Zn (30 mg/kg b.w.) according to Partyka et al. [36] and Anna Partyka et al. [37] orally for 3 weeks in drinking water once per day and rotenone (2.5 mg/kg) once a day for the last seven consecutive days. Group IV received a daily dose of Linoleic acid (150 µg/kg) subcutaneously for 3 weeks once per day and rotenone (2.5 mg/kg) once a day for the last seven consecutive days. Group V received both Zinc (30 mg/kg) and Linoleic acid (150 µg/kg) orally and subcutaneously once per day for 3 weeks respectively and rotenone (2.5 mg/kg) once a day for the last seven consecutive days. Group VI served as a positive control and received Levodopa (6 mg/kg) orally once per day with rotenone (2.5 mg/kg) once a day consecutively for 7 days.

### Brain tissue processing

At the end of the experiment, the rats were fasted overnight and humanely sacrificed by cervical dislocation without anaesthesia. The whole brain of each rat was rapidly dissected, removed and rinsed in ice-cold isotonic saline. The whole brain was weighed and immediately the midbrain region was isolated and this isolated brain section was used for further investigations. Portions of the midbrain were removed to use for histological assessments. The remainder midbrain tissues were homogenized with ice-cold 0.1 M phosphate buffer saline (pH 7.4). Tissue homogenate (10% w/v) was prepared in 50 mM phosphate buffer saline (pH 7.4) using a Symphony type Eperndorff homogenizer. The brain homogenate was centrifuged at 2000*g* for 10 min at 4 °C. The pellet which contained debris and nuclei was discarded. The supernatant was then again centrifuged at 12,000*g* for 20 min to obtain post mitochondrial supernatant. The supernatant was kept at − 80 °C until determination of the levels and activity of oxidative stress markers [25].

### Brain oxidative stress marker assessments

#### Brain lipid peroxidation

The method described by Stocks et al. [47] was used to measure lipid peroxidation. A coloured complex called thiobarbituric acid reactive substance is formed from a reaction of malondialdehyde and thiobarbituric acid. The complex was assayed spectrophotometrically at 532 nm and expressed in micro moles.

#### Brain total antioxidant capacity

The Ferric Reducing Antioxidant Power (FRAP) was used as a method of measuring the total antioxidant capacity of the homogenate [4]. This method determines the capacity of the homogenate to reduce ferric iron to ferrous iron at a pH of 3.6. A deep blue coloured compound of ferrous tripyridyltriazine (TPTZ-Fe-2+) is formed as a result of the reduction of ferric tripyridyltriazide (TPTZ-Fe3+). The absorbance was measured at 593 nm and the results were expressed in micromolar.

#### Brain reduced glutathione

The method of Boyne and Ellman [6] was used to estimate the level of reduced GSH in the brain homogenate. To precipitate the tissue proteins in the homogenate, it was mixed with 0.1 M phosphate buffer (pH 7.4) and then added to equal volume of 20% trichloroacetic acid containing 1 mM EDTA. The mixture stood for 5 min before it was centrifuged for 10 min at 2000 rpm. The supernatant was then transferred to a new set of test tubes, to which was added 1.8 ml of the Ellman’s reagent (5,5′-dithio bis-2-nitrobenzoic acid). The reaction mixture was measured at 412 nm against blank.

#### Brain superoxide dismutase activity

The method of Misra and Fridovich [32] was used to assay the activity of superoxide dismutase. The method is based on the principle that super oxide inhibits the oxidation of adrenaline to adrenochrome. Each 3-ml of the reaction mixture contained 0.1-ml tissue homogenate, 2.8 ml of Potassium phosphate buffer (0.1 M, pH 7.4), and 0.1-ml pyrogallol solution (2.6 mM in 10 mM HCl). The pink product formed, adenochrome was detected in a spectrophotometer at 480 nm. The results were expressed as units/mg protein.

#### Brain catalase activity

The method of Sinha [45] was used to assay catalase activity. A volume of 0.5 ml of the homogenate was added to the reaction mixture which contained 1 ml of 0.01 M phosphate buffer (pH 7.0), 0.5 ml of 0.2 M H_2_O_2_, and 0.4 ml H_2_O. The tubes were all heated at 95 °C for 10 min. To terminate the reaction, 2 ml of dichromate/acetic acid mixture was added to it. To the control, the homogenate was added after the addition of acid reagent. The absorbance was read at 570 nm, the enzyme activity was expressed as micromoles of H_2_O_2_/min/mg of protein.

#### Brain glutathione peroxidase

A colorimetric assay kit was used to assay the activity of glutathione peroxidase (GPx) (Sigma-Aldrich, Germany). This test is based on the principle that reduction of organic peroxide by glutathione peroxidase produces oxidized glutathione which is immediately converted to its reduced form (GSH) at the same time oxidizing NADPH to NADP+. The oxidation of NADPH was monitored spectrophotometrically using as a decrease in absorbance at 340 nm [14].

#### Total brain protein

Quantitative estimation of brain homogenate total protein was carried out according to the Biuret method Gornall et al. [17]. This was done in order to quantify the concentration of proteins in the samples.

### Light microscopic examination

Small sections of each midbrain from the normal control and the various treated animals were fixed on 10% paraformaldehyde to assess for histological changes. Thin frozen brain sections of 30 µm from were obtained using microtome. The tissues were dehydrated in ascending grades of alcohol following fixation, and were then embedded in wax. Approximately 5–7 µm thick paraffin sections were cut and then subjected to hematoxylin–eosin staining as described by Fischer et al. [11]. Appearances of necrosis, apoptosis, size and quantity of cells were observed. The prepared slides were sent to the histopathology Department of the University of Yaounde, 1 Teaching Hospital for specialist observation and interpretation.

### Statistical analysis

Results were collected, tabulated and expressed as mean ± S.E.M. Measurements were analyzed using one-way analysis of variance, ANOVA, followed by Tukey’s multiple comparisons test. All statistical tests were done employing Graph Pad Prism version 6. Differences were considered significant at p ≤ 0.05.

## Results

In this present study, subcutaneous administration of rotenone (2.5 mg/kg) to rats consecutively for 7 days produced biochemical alterations accompanied by histological changes in the neurons in the midbrain.

### Percentage survival

Repeated treatment of rats in this study with rotenone resulted in morbidity and mortality in rats. Death began occurring from the 4th day of administration of rotenone. The percentage survival of rats at the end of the experiment was found to reach 83% (5 out of 6). Death of rats occurred in the rotenone (01), zinc and Linoleic (01) and levodopa group (01). The survival of rats was not improved by treatment with Zinc and Linoleic acid as compared to rotenone group. From day 3, some animals showed severe weakness, apparent weight loss and locomotive inability. Rats that died during the course of the experiment were excluded from statistical analysis.

### Biochemical measurements

#### Lipid peroxidation by MDA measurement

**Fig. 1:**
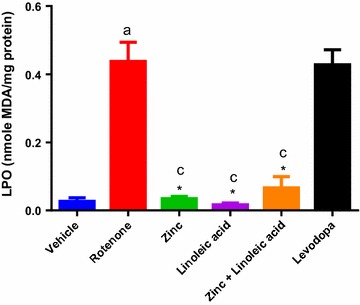
Lipid peroxidation (MDA) levels in the midbrain of experimental groups. ^a^p ≤ 0.0001 compared to vehicle group, ^c^p ≤ 0.0001 compared to levodopa group, *p ≤ 0.0001 compared to rotenone group

In the estimation of MDA levels, all animals in each group showed variable levels of MDA (Fig. 1 and Table 1). There was significant increase in levels of MDA in rotenone treated animals (p < 0.0001), when compared with normal control animals (0.03 ± 0.01 to 0.44 ± 0.06). There was significant decrease in MDA levels in Zinc treated animals (0.44 ± 0.06 to 0.03 ± 0.01; p < 0.0001), Linoleic acid (0.44 ± 0.06 to 0.02 ± 0.00; p < 0.0001), and a combination of both (0.44 ± 0.06 to 0.07 ± 0.03; p < 0.0001) when compared with rotenone treated animals. The effect of the zinc, Linoleic acid and the combination was significantly greater when compared to the Levodopa group (p < 0.0001 Table 1). There was however no significant difference in MDA levels of the combination when compared with individual treatments. Zinc lowered the MDA levels to normal control group levels (0.03 ± 0.01 to 0.03 ± 0.01), while Linoleic acid and the combination groups lowered to near normal control levels (0.03 ± 0.01 to 0.02 ± 0.00 and 0.03 ± 0.01 to 0.07 ± 0.03) respectively. Levodopa did not significantly reduce the MDA levels (0.44 ± 0.06 to 0.43 ± 0.04, p > 0.05, Table 1). The effect of linoleic acid and the combination was significant when compared to levodopa (p < 0.0001).

**Table 1:** Effect of zinc and linoleic acid and their combination on LPO and the antioxidant system enzymes in mid brain of rotenone treated rats

Groups	MDA (µmoles/mg protein)	TAC (µmoles/mg protein)	GSH (µmoles/mg protein)	SOD (UI/mg protein)	CAT (µmole H_2_O_2_/min/mg protein)	GPx (UI/ml enzymes)	TP (mg protein)
	Mean ± SEM						
Vehicle (n = 6)	0.03 ± 0.01	699.2 ± 3.473	4.44 ± 0.43	6.68 ± 1	0.64 ± 0.01	0.0200 ± 0.003536	10.67 ± 1.052
Rotenone (n = 5)	0.44 ± 0.06^a^	554.6 ± 8.921^a^	0.57 ± 0.18^a^	1.42 ± 0.63^a^	0.22 ± 0.03^a^	0.0062 ± 0.002131	9.370 ± 0.3590
Zinc (n = 6)	0.03 ± 0.01*^c^	686.7 ± 8.559*	3.91 ± 0.31*	5.16 ± 0.24^b^	0.74 ± 0.03*^,d^	0.0358 ± 0.006778^b^	10.41 ± 0.06512
Linoleic acid (n = 6)	0.02 ± 0.00*^c^	702.1 ± 4.699*	3.76 ± 0.49*	5.34 ± 1.04^b^	0.74 ± 0.04*^,d^	0.0752 ± 0.004758*^,c^	10.95 ± 0.2067
Zinc + linoleic acid (n = 5)	0.07 ± 0.03*^c^	630.2 ± 33.28^b^	3.62 ± 0.34*	7.11 ± 0.73^b^	0.68 ± 0.05*	0.0812 ± 0.007716*^,c^	11.30 ± 0.6652
Levodopa (n = 5)	0.43 ± 0.04	692.7 ± 3.239*	3.69 ± 0.23*	5.09 ± 0.55^b^	0.50 ± 0.08^b^	0.0170 ± 0.0030	11.28 ± 0.3937

Total protein and antioxidant biomarker activity and concentration in the midbrain of the experimental groups. Results are expressed as mean ± SEM

*LPO* lipid peroxidation

^a^p ≤ 0.0001 compared to vehicle group; ^b^p ≤ 0.05 compared to rotenone group; ^c^p ≤ 0.0001 compared to levodopa group, ^d^p ≤ 0.05 compared to levodopa group

*p ≤ 0.0001 compared to rotenone group

#### Total antioxidant capacity

**Fig. 2:**
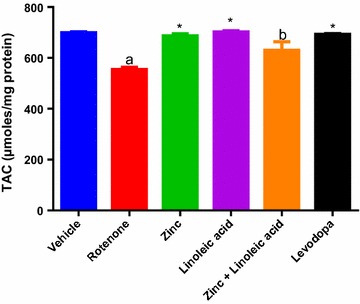
Total antioxidant capacity (TAC) levels in the midbrain of experimental groups. ^a^p ≤ 0.0001 compared to vehicle group, ^b^p ≤ 0.05 compared to rotenone group, *p ≤ 0.0001 compared to rotenone group

The total antioxidant capacity in rotenone-treated rats presented in the Fig. 2 and Table 1 was found to significantly (p ≤ 0.05) decrease compared to vehicle-treated rats (699.2 ± 3.473 to 554.6 ± 8.921). The zinc and linoleic acid groups had significantly prevented the decrease in total antioxidant capacity (TAC) caused by rotenone administration (554.6 ± 8.921 to 686.7 ± 8.559; 554.6 ± 8.921 to 702.1 ± 4.699 respectively) (p < 0.0001); their combination also prevented this decrease in TAC caused by rotenone administration (554.6 ± 8.921 to 630.2 ± 33.28) (p ≤ 0.05, Fig. 2). The effect of the combination was not significant when compared to single treatments (p > 0.05, Fig. 2). Zinc, linoleic acid, the combination and levodopa all increased TAC to near normal control levels.

#### Reduced glutathione concentration

**Fig. 3:**
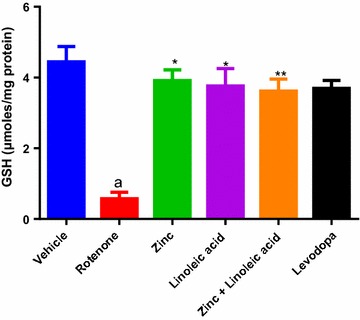
Reduced glutathione levels in the midbrain of experimental groups. ^a^p ≤ 0.05 compared to vehicle group, *p ≤ 0.0001 compared to rotenone group

There was significant decrease of reduced glutathione (GsH) level (4.44 ± 0.43 to 0.57 ± 0.18) in brain content in rotenone treated groups as compared to the vehicle group (p ≤ 0.0001) (Table 1 and Fig. 3). There was significant increase in reduced glutathione levels in zinc treated animals i.e. 0.57 ± 0.18 to 3.91 ± 0.31 (p < 0.0001), linoleic acid 0.57 ± 0.18 to 3.76 ± 0.49 (p < 0.0001), and a combination of both i.e. 0.57 ± 0.18 to 3.62 ± 0.34 (p < 0.0001) when compared with rotenone treated animals i.e. 0.57 ± 0.18. The combination of zinc and linoleic acid did not significantly increase GSH levels when compared to individual treatment (p > 0.05, Table 1, Fig. 3). Levodopa significantly increased brain GSH levels compared to the rotenone group (0.57 ± 0.18 to 3.69 ± 0.23). The effect of zinc, linoleic acid, their combination and levodopa was not significant compared to the normal control group.

#### Superoxide dismutase

**Fig. 4:**
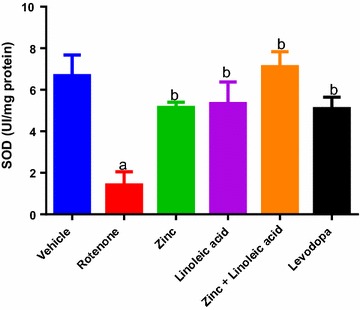
Superoxide dismutase level in the midbrain of the experimental groups. ^a^p ≤ 0.05 compared to vehicle group, ^b^p ≤ 0.05 compared to rotenone group

There was a significant decrease in SOD activity in rotenone-treated rats as compared to vehicle-treated rats (6.68 ± 1 to 1.42 ± 0.63; p ≤ 0.05, Fig. 4 and Table 1). The zinc group had a significant increase in SOD activity as compared to rotenone group (1.42 ± 0.63 to 5.16 ± 0.24, p < 0.05). The linoleic acid group showed a similar effect (1.42 ± 0.63 to 5.34 ± 1.04, p < 0.05), as did their combination enhanced SOD activity as compared to rotenone group (1.42 ± 0.63 to 7.11 ± 0.73, p ≤ 0.05, Fig. 4). The combination of zinc and linoleic acid also significantly increased SOD activity when compared to individual treatment (p < 0.05, Fig. 4). The effect of zinc, linoleic acid, their combination and levodopa was not significant when compared to the normal control group. Also the effect of zinc, linoleic acid and their combination was not significant when compared to levodopa (p > 0.05).

#### Glutathione peroxidase

**Fig. 5:**
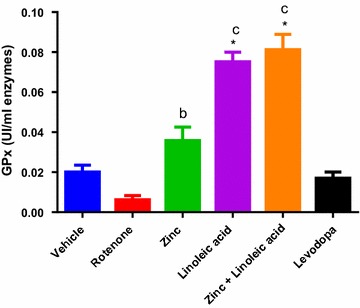
Glutathione peroxidase activity in the midbrain of experimental groups. ^b^p ≤ 0.05 compared to rotenone group, ^c^p ≤ 0.0001 compared to levodopa group, *p ≤ 0.0001 compared to rotenone group

The glutathione peroxidase activity was not significantly decreased in rotenone-treated rats as compared to vehicle-treated rats (0.0200 ± 0.003536 to 0.0062 ± 0.002131 p > 0.05, Fig. 5 and Table 1). The linoleic acid and the combination group had significant increase in GPx activity as compared to rotenone group (0.0062 ± 0.002131 to 0.0752 ± 0.004758 and 0.0062 ± 0.002131 to 0.0812 ± 0.007716 respectively, p < 0.0001, Table 1); the zinc group also showed a significant effect (0.0062 ± 0.002131 to 0.0358 ± 0.006778, p ≤ 0.05, Fig. 5). The effects of zinc and linoleic acid on increasing GPx activity were significant in comparison to the normal control group (0.0200 ± 0.003536 to 0.0358 ± 0.006778 and 0.0200 ± 0.003536 to 0.0752 ± 0.004758 respectively, p < 0.0001). The effects of linoleic acid and the combination was significant when compared to levodopa (p < 0.0001) while zinc effect was not significant (p > 0.05).

#### Catalase

**Fig. 6:**
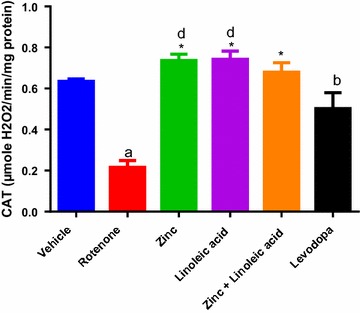
Catalase activity in the midbrain of experimental groups. ^a^p ≤ 0.0001 compared to vehicle group, ^b^p ≤ 0.05 compared to rotenone group, ^d^p ≤ 0.05 compared to levodopa group, *p ≤ 0.0001 compared to rotenone group

Catalase activity in the vehicle treated group was found to be 0.64 ± 0.01 μM of H_2_O_2_ used/min/mg protein (Fig. 6). Rotenone treatment resulted in a significant decrease in CAT level in the midbrain as compared to the vehicle-treated group (0.64 ± 0.01 to 0.22 ± 0.03, p < 0.0001). The zinc group (0.22 ± 0.03 to 0.74 ± 0.03), linoleic acid group (0.22 ± 0.03 to 0.74 ± 0.04) and their combination (0.22 ± 0.03 to 0.68 ± 0.05) had a significant increase in CAT activity as compared to rotenone group; (p ≤ 0.0001, Fig. 6). The combination of zinc and linoleic acid however did not significantly increase catalase activity when compared to individual treatment (p > 0.05, Table 1, Fig. 6). The CAT activity increasing effects of zinc, Linoleic acid and their combination was not significant when compared to the normal control and levodopa groups.

### Histopathology

**Fig. 7:**
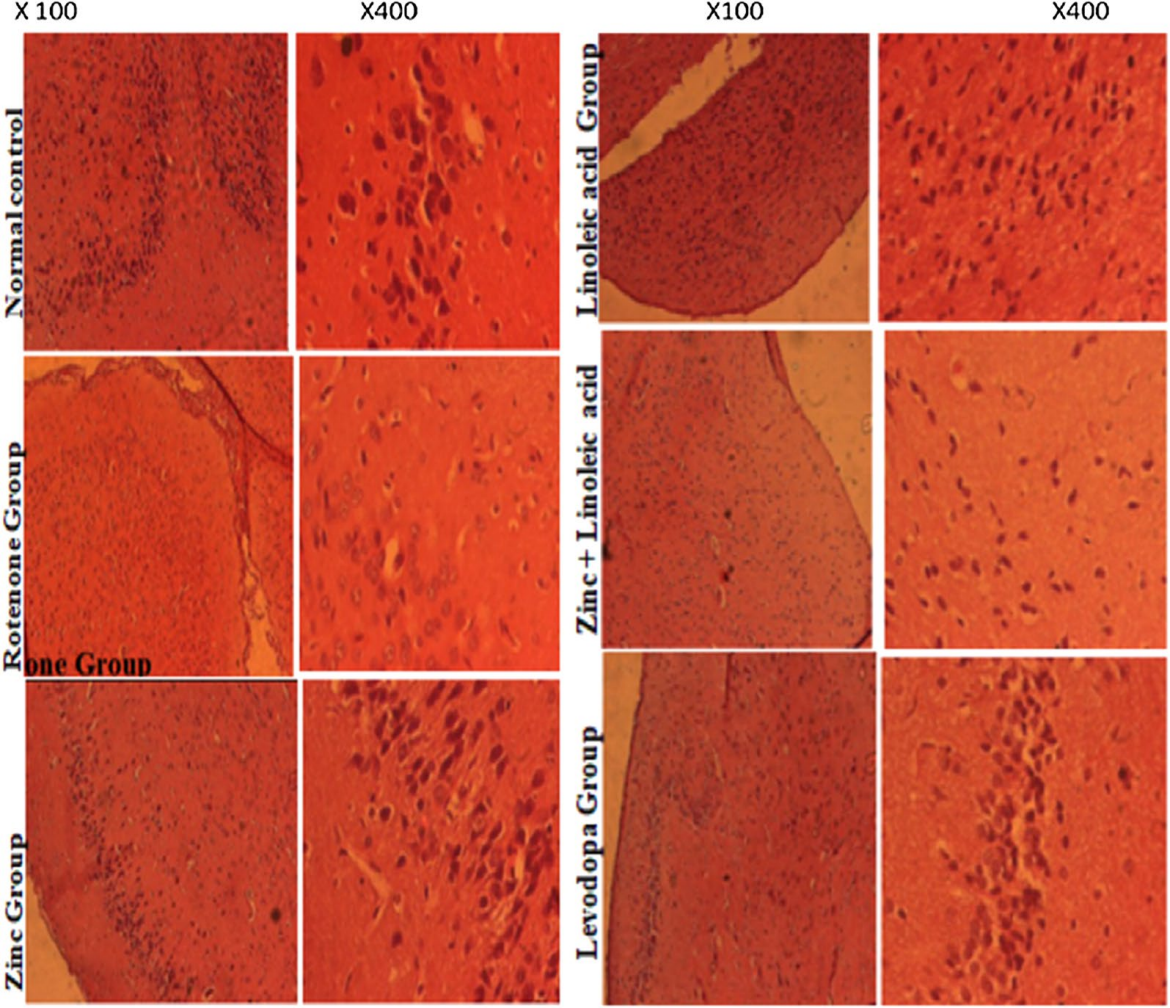
Histopathological changes in the mid brain of rats stained with haematoxylin and eosin ×100 and ×400: vehicle section showing normal histoarchitecture. Rats treated with rotenone (2.5 mg/kg) with decrease neuron size and density. Rats treated with rotenone and interventions showing increased cellularity compared to rotenone group alone

The vehicle-treated group animals showed normal neuronal density and normal cellularity of neurons with no atypical cells, no visible cell death (Fig. 7). In rotenone treated group there was neuronal cell death, slight structural damage to the midbrain. The zinc group showed midbrain structure with slight hyperplasia of cells, presence of micro vacuolization and augmented number of cells reflecting cellular injury however with small blood vessels. There was no visible cell death in the linoleic acid group. The combination group showed normal neuron cell population, with some cells appearing multinucleated. The levodopa group showed slight cellularity of the midbrain region with multinucleated cells. There was no apparent cell death.

## Discussion

The present study was done to evaluate the role of Zinc and Linoleic acid and their combination, as neuroprotective agents against rotenone-induced parkinsonism. As shown previously [2] intraperitoneal injection of rotenone over 7 days precipitates biochemical and histological deficits in the midbrain of rats. Interestingly, pre-treatment of rats with Zinc or Linoleic acid prevented the decrease in total brain antioxidant capacity and activities of antioxidant enzymes.

In animal model studies, Rotenone (an alkaloidal pesticide), a specific inhibitor of complex I is employed to increase oxidative stress-mediated neuropathology [50]. More specifically, it is used to generate toxin-based rodent models of Parkinson’s disease [43]. Because of its mechanism of action involving oxidative stress, exposure of rodents to Rotenone provides a valuable model for studying both mechanisms of oxidative stress and neuroprotection by antioxidant agents [39]. Pathologically, Rotenone induces the degeneration of the nigrostriatal dopaminergic pathway which is one of the cardinal pathological markers of Parkinson’s disease [2, 50].

A number of studies have shown that the dopaminergic neurons in PD exists in a state of constant oxidative stress, due in part largely to the generation of H_2_O_2_ [24]. Lipid peroxidation measured by MDA levels was observed to be elevated in rotenone treated animals than controls. This agrees with other studies which suggest that oxidative damage is involved in the neuronal abnormalities in PD [28, 53]. However, treatment with zinc and linoleic acid or their combination reduced MDA levels and therefore lipid peroxidation and brought it to control levels. The reduction of MDA levels and thus lipid peroxidation by zinc is in line with the study of Ozturk et al. [34] who investigated the effects of zinc deficiency and supplementation on malondialdehyde and glutathione levels in blood and tissues of rats performing swimming exercise, and showed that zinc supplemented rats had increased reduced glutathione and decreased MDA levels. The lowered MDA levels due to linoleic acid corroborates with the studies of Yang and colleagues who showed reduced MDA levels in the hippocampus of diabetic rats on administration of omega-3 PUFA [55]. The mechanism involved in lowering lipid peroxidation by linoleic acid appears to involve detoxification of peroxy radicals and other ROS species [16].

Decreased levels of GSH might play an important role in inducing oxidative stress in brain [57]. Such levels have been detected in remaining neurons and in SNpc of PD patients as compared to controls of similar age [49]. In this present study, GSH levels were lower in rotenone treated animals. Studies have shown decreased levels of GSH leads to oxidative damage to DNA, protein and lipids in PD [8, 48, 54]. Oral administration of zinc and subcutaneous administration of Linoleic acid increased GSH levels. Zinc has been suggested to exert this GSH-increased level effect through the actions of metallothionein [40]. On the other hand the GSH activity-increasing effect of linoleic acid corroborates with the study of Ahmad and Beg [1], who evaluated the therapeutic effect of omega-6 linoleic acid and thymoquinone enriched extracts from Nigella sativa oil in the mitigation of lipidemic oxidative stress in rats. The effects of linoleic acid on GSH may be due to their direct antioxidant effects or the prevention of GSH oxidation.

The rotenone treated group showed a significant decrease in glutathione peroxidase activity. This agrees with the studies of Testa et al. [51]. Zinc pre-treatment was able to increase glutathione peroxidase activity. This corroborates with the study of the effect of zinc supplementation on glutathione peroxidase activity and selenium concentration in the serum, liver and kidney of rats chronically exposed to cadmium by Galażyn-Sidorczuk et al. [14], showed that zinc significantly increased glutathione peroxidase levels. Linoleic acid in the study of Ahmad and Beg [1] also protected glutathione peroxidase activity by 90%.

SOD activity in the group treated with rotenone was found to be reduced. Complex-I of the mitochondrial respiratory chain is a major source of superoxide free radicals. The loss in SOD activity might contribute to increase in oxidative stress in rotenone treated animals [12, 27]. A lowered SOD activity would be detrimental in cases when superoxide radical production is increased. The decrease in SOD activity following rotenone treatment might be due to inactivation of SOD by ROS [12]. Administration of zinc was beneficial in restoring the SOD activity. This agrees with the studies of Paz Matias et al. [38] who investigated the effect of zinc supplementation on superoxide dismutase activity in patients with ulcerative rectocolitis. In the study of Ahmad and Beg [1], omega-6 linoleic acid supplementation also increased SOD activity.

The present study showed reduced CAT activity in rotenone-treated rats as compared to vehicle-treated rats. This agrees with the studies of Liu et al. [26], Prema et al. [41] and Soczynska et al. [46]. A study by Sharma and Bafna [42] showed a non-significant increase in the activity of CAT in rotenone treated rats compared to vehicle-treated rats. Zinc and linoleic acid increased the activity of CAT compared to the rotenone treated group. Zinc effect on Catalase activity agrees on the study of [35] who investigated the effect of zinc supplementation on antioxidant status and immune response in buffalo calves and showed that zinc supplementation increased Catalase activity. The protective effects of linoleic acid might reflect its ability to improve energy metabolism and repair damaged layers of lipids, hence suppressing the exudation of free electrons from the mitochondrial electron transport system, which is a prerequisite reaction to generate free radicals [29].

The antioxidant effect of Zinc has been suggested to work via metallothionein by regulating the secretion of pro-inflammatory cytokines and these metallothionein’s are strong scavengers of free radicals [20, 31]. Linoleic acid with efficient free radical scavenging capacity could be involved in lowering MDA and slowing the degradation of the antioxidant enzymes CAT, SOD and GPx on its administration [9, 23], thus improving the total antioxidant capacity. The combination of zinc and linoleic acid showed remarkable antioxidant effect by lowering MDA levels and increasing GSH, SOD above the levels of individual interventions. Zinc and Linoleic acid antioxidant activity from the combination group did not show additive effect, supporting cited literature that Zinc and Linoleic acid act through independent pathways. We however suggest that either of the two facilitate the activity of the other.

The Levodopa treated group showed a significant increase in the total antioxidant capacity, levels and activity of antioxidant markers (Table 1) compared to the rotenone treated group. This corroborates with previous studies of Testa et al. [51], who evaluated the Levodopa and carbidopa antioxidant activity in normal lymphocytes in vitro, examining their implication for oxidative stress in Parkinson’s disease. Their studies showed that Levodopa protected DNA from damage, scavenged free radicals and modulated the expression of genes involved in cellular oxidative metabolism.

In histopathological findings visible neuronal cell loss in the midbrain was identified that indicates the damage of neurons at this region in rotenone treated animals. Findings from earlier studies have shown that the substantia nigra is more vulnerable to damage by rotenone [26]. This may be attributed to higher susceptibility of the neurons in this region to free radicals. Histological findings in the midbrain were more correlated with impaired motor coordination responses along with biochemical evidences [51]. This may indicate that the midbrain area responds highly to oxidative stress caused by rotenone. This vulnerability of the midbrain to rotenone-induced oxidative stress has also been demonstrated in other studies [43]. Zinc and linoleic acid and their combination improved the neuronal loss in the midbrain caused by rotenone administration. The decreases in necrotic cells observed in the zinc treated group agrees with the studies of Galvão et al. [15] who also observed a decrease in the number of necrotic cells in hippocampus as well as cortex of zinc supplemented group for both male and female pups, in a study investigating the effect of “Prenatal zinc in adult rat offspring exposed to lipopolysaccharide during gestation”. Linoleic acid effect on the midbrain corroborates with the studies of Beltz et al. [3], who investigated the effect of Linoleic acid on hippocampal neurogenesis. They advanced suggestions that linoleic acid plays this role via increasing expression of brain derived neurotrophic factor. The group treated with Levodopa were protected from neuronal loss in the midbrain region.

## Limitations

Even though the study did not set out to investigate sex difference or confounding role of female sex hormones, the use of female rats in this study was a matter of choice motivated by the limited studies conducted on the effect of these micronutrients and trace elements on female rats. It is worth noting that the impact of the differences in estrous cycles of the rats could not be accounted for. It has been shown that estrous cycle variation, a consequence of variation in the levels of reproductive hormones, also influence the expression of genes responsible for production of antioxidants [32]. Our experimental design did not consider the impact of this variation on the results.

## Conclusion

In the present study, repeated systemic administration of rotenone (2.5 mg/kg doses, s.c.) in rats produced increased midbrain lipid peroxidation and impaired antioxidant status, accompanied by histological changes. Zinc, Linoleic acid and their combination prevented the increase in MDA levels and decrease in brain antioxidant status induced by rotenone treatment. Cell death and reduction in neuron size induced by rotenone was prevented by treatment with zinc, linoleic acid and their combination. However further studies are needed to explore the possible mechanisms involved in this behavioral effect.

## Abbreviations

CAT: Catalase
DMSO: Dimethylsulfoxide
DAergic: Dopaminergic
G-Px: Glutathione peroxidase
MDA: Malondialdehyde
PD: Parkinson’s disease
ROS: Reactive oxygen species
PBS: Phosphate-buffered saline
PUFAs: Poly-unsaturated fatty acids
GSH: Reduced glutathione
SNpc: Substantia nigra pars compacta
SOD: Superoxide dismutase

## References

[1] Ahmad S, Beg ZH (2016). Evaluation of therapeutic effect of omega-6 linoleic acid and thymoquinone enriched extracts from Nigella sativa oil in the mitigation of lipidemic oxidative stress in rats. Nutrition.

[2] Alam M, Schmidt WJ (2002). Rotenone destroys dopaminergic neurons and induces parkinsonian symptoms in rats. Behav Brain Res.

[3] Beltz BS, Tlusty MF, Benton JL, Sandeman DC (2007). Omega-3 fatty acids upregulate adult neurogenesis. Neurosci Lett.

[4] Benzie I, Strain J (1996). The ferric reducing ability of plasma (FRAP) as a measure of “antioxidant power”: the FRAP assay. Anal Biochem.

[5] Blesa J, Trigo-Damas I, Quiroga-Varela A, Jackson-Lewis VR (2015). Oxidative stress and Parkinson’s disease. Front Neuroanat.

[6] Boyne AF, Ellman GL (1972). A methodology for analysis of tissue sulfhydryl components. Anal Biochem.

[7] Calon F, Cole G (2007). Neuroprotective action of omega-3 polyunsaturated fatty acids against neurodegenerative diseases: evidence from animal studies. Prostaglandins Leukot Essent Fat Acids.

[8] Chinta SJ, Andersen JK (2008). Redox imbalance in Parkinson’s disease. Biochim Biophys Acta Gen Subj.

[9] Eckert GP, Lipka U, Muller WE (2013). Omega-3 fatty acids in neurodegenerative diseases: focus on mitochondria. Prostaglandins Leukot Essent Fat Acids.

[10] Elbaz A, Tranchant C (2007). Epidemiologic studies of environmental exposures in Parkinson’s disease. J Neurol Sci.

[11] Fischer AH, Jacobson KA, Rose J, Zeller R (2005). Hematoxylin and eosin (H & E) staining. CSH Protoc.

[12] Fridovich I (1995). Superoxide radical and superoxide dismutases. Ann Rev Biochem.

[13] Fujikawa T, Kanada N, Shimada A, Ogata M, Suzuki I, Hayashi I, Nakashima K (2005). Effect of sesamin in Acanthopanax senticosus HARMS on behavioral dysfunction in rotenone-induced parkinsonian rats. Biol Pharm Bull.

[14] Galażyn-Sidorczuk M, Brzóska MM, Rogalska J, Roszczenko A, Jurczuk M (2012). Effect of zinc supplementation on glutathione peroxidase activity and selenium concentration in the serum, liver and kidney of rats chronically exposed to cadmium. J Trace Elem Med Biol.

[15] Galvão MC, Chaves-Kirsten GP, Queiroz-Hazarbassanov N, Carvalho VM, Bernardi MM, Kirsten TB (2015). Prenatal zinc reduces stress response in adult rat offspring exposed to lipopolysaccharide during gestation. Life Sci.

[16] Gladine C, Newman JW, Durand T, Pedersen TL, Galano JM, Demougeot C, Comte B (2014). Lipid profiling following intake of the omega 3 fatty acid DHA identifies the peroxidized metabolites F4-neuroprostanes as the best predictors of atherosclerosis prevention. PLoS ONE.

[17] Gornall AG, Bardawill CJ, David MM (1949). Determination of serum proteins by means of the biuret reaction. J Biol Chem.

[18] Hallett M (2014). Tremor: pathophysiology. Parkinsonism Relat Disord.

[19] Henchcliffe C, Beal MF (2008). Mitochondrial biology and oxidative stress in Parkinson disease pathogenesis. Nat Clin Pract Neurol..

[20] Hijova E (2004). Metallothioneins and zinc: their functions and interactions. Bratisl Lekarske Listy.

[21] Hirose G (2006). Drug induced parkinsonism. J Neurol.

[22] Hwang O (2013). Role of oxidative stress in Parkinson’s disease. Exp Neurobiol.

[23] Innis SM (2008). Dietary omega 3 fatty acids and the developing brain. Brain Res.

[24] Jenner P, Olanow CW (1996). Oxidative stress and the pathogenesis of Parkinson’s disease. Neurology.

[25] Kaur H, Chauhan S, Sandhir R (2011). Protective effect of lycopene on oxidative stress and cognitive decline in rotenone induced model of Parkinson’s disease. Neurochem Res.

[26] Lehto SM, Ruusunen A, Tolmunen T, Voutilainen S, Tuomainen TP, Kauhanen J (2013). Dietary zinc intake and the risk of depression in middle-aged men: a 20-year prospective follow-up study. J Affect Disord..

[27] Liochev SI, Fridovich I (2007). The effects of superoxide dismutase on H_2_O_2_ formation. Free Radic Biol Med.

[28] Liu CB, Wang R, Pan HB, Ding QF, Lu FB (2013). Effect of lycopene on oxidative stress and behavioral deficits in rotenone induced model of Parkinson’s disease. Zhongguo Ying Yong Sheng Li Xue Za Zhi..

[29] Liu J, Killilea DW, Ames BN (2002). Age-associated mitochondrial oxidative decay: improvement of carnitine acetyltransferase substrate-binding affinity and activity in brain by feeding old rats acetyl-l-carnitine and/or R-alpha-lipoic acid. Proc Natl Acad Sci USA.

[30] Luchtman DW, Meng Q, Song C (2012). Ethyl-eicosapentaenoate (E-EPA) attenuates motor impairments and inflammation in the MPTP-probenecid mouse model of Parkinson’s disease. Behav Brain Res.

[31] Maret W (2013). Zinc and human disease. Metal Ions Life Sci.

[32] Misra HP, Fridovich I (1972). The role of superoxide anion in the autoxidation of epinephrine and a simple assay for superoxide dismutase. J Biol Chem.

[33] Ojha S, Javed H, Azimullah S, Khair SBA, Haque ME (2015). Neuroprotective potential of ferulic acid in the rotenone model of Parkinson’s disease. Drug Des Dev Ther.

[34] Ozturk A, Baltaci AK, Mogulkoc R, Oztekin E, Sivrikaya A, Kurtoglu E, Kul A (2003). Effects of zinc deficiency and supplementation on malondialdehyde and glutathione levels in blood and tissues of rats performing swimming exercise. Biol Trace Elem Res.

[35] Parashuramulu S, Nagalakshmi D, Rao DS, Kumar MK, Swain PS (2015). Effect of zinc supplementation on antioxidant status and immune response in buffalo calves. Anim Nutr Feed Technol.

[36] Partyka A, Jastrzebska-Wiesek M, Nowak G. Evaluation of anxiolytic-like activity of zinc. In: 17th international congress of the polish pharmacological society Krynica Zdroj Poland; 2010. vol. 62, pp. 57–58.

[37] Partyka A, Jastrzȩbska-Wiȩsek M, Szewczyk B, Stachowicz K, Sałwinśka A, Poleszak E, Nowak G (2011). Anxiolytic-like activity of zinc in rodent tests. Pharmacol Rep.

[38] Paz Matias J, Costa e Silva DM, Climaco Cruz KJ, Gomes da Silva K, Monte Feitosa M, Oliveira Medeiros LG, do Nascimento Nogueira N (2015). Effect of zinc supplementation on superoxide dismutase activity in patients with ulcerative rectocolitis. Nutr Hosp.

[39] Perfeito R, Cunha-Oliveira T, Rego AC (2012). Revisiting oxidative stress and mitochondrial dysfunction in the pathogenesis of Parkinson disease–resemblance to the effect of amphetamine drugs of abuse. Free Radic Biol Med.

[40] Prasad AS (2014). Zinc: an antioxidant and anti-inflammatory agent: role of zinc in degenerative disorders of aging. J Trace Elem Med Biol.

[41] Prema A, Janakiraman U, Manivasagam T, Arokiasamy JT (2015). Neuroprotective effect of lycopene against MPTP induced experimental Parkinson’s disease in mice. Neurosci Lett.

[42] Sharma N, Bafna P (2012). Effect of Cynodon dactylon on rotenone induced Parkinson’s disease. Orient Pharm Exp Med.

[43] Sherer TB, Betarbet R, Testa CM, Seo BB, Richardson JR, Kim JH, Greenamyre JT (2003). Mechanism of toxicity in rotenone models of Parkinson’s disease. J Neurosci.

[44] Shin HW, Chung SJ (2012). Drug-Induced parkinsonism. J Clin Neurol (Korea).

[45] Sinha AK (1972). Colorimetric assay of catalase. Anal Biochem.

[46] Soczynska JK, Kennedy SH, Chow CS, Woldeyohannes HO, Konarski JZ, McIntyre RS (2008). Acetyl-l-carnitine and α-lipoic acid: possible neurotherapeutic agents for mood disorders?. Expert Opin Investig Drugs.

[47] Stocks J, Gutteridge JMC, Sharp RJ, Dormandy TL (1974). The inhibition of lipid autoxidation by human serum and its relation to serum proteins and α to copherol. Clin Sci Mol Med.

[48] Surapaneni K, Venkataramana G (2007). Status of lipid peroxidation, glutathione, ascorbic acid, vitamin E and antioxidant enzymes in patients with osteoarthritis. Indian J Med Sci.

[49] Tamilselvam K, Braidy N, Manivasagam T, Essa MM, Prasad NR, Karthikeyan S, Guillemin GJ (2013). Neuroprotective effects of hesperidin, a plant flavanone, on rotenone-induced oxidative stress and apoptosis in a cellular model for Parkinson’s disease. Oxidative Med Cell Longev.

[50] Tanner et al. Rotenone, paraquat, and Parkinson’s disease. Environ Health Perspect. 2011;119(6):866–72. http://www.sertox.com.ar/modules.php?name=News&file=article&sid=3681. 10.1289/ehp.1002839 PMC3114824 21269927

[51] Testa CM, Sherer TB, Greenamyre JT (2005). Rotenone induces oxidative stress and dopaminergic neuron damage in organotypic substantia nigra cultures. Mol Brain Res.

[52] Thiffault C, Langston JW, Di Monte DA (2000). Increased striatal dopamine turnover following acute administration of rotenone to mice. Brain Res..

[53] Tsang AHK, Chung KKK (2009). Oxidative and nitrosative stress in Parkinson’s disease. Biochem Biophys Acta.

[54] Vinish M, Anand A, Prabhakar S (2011). Altered oxidative stress levels in Indian Parkinson’s disease patients with PARK2 mutations. Acta Biochim Pol.

[55] Yang R-H, Wang F, Hou X-H, Cao Z-P, Wang B, Xu X-N, Hu S-J (2012). Dietary ω-3 polyunsaturated fatty acids improves learning performance of diabetic rats by regulating the neuron excitability. Neuroscience.

[56] Zawada WM, Banninger GP, Thornton J, Marriott B, Cantu D, Rachubinski AL, Jones SM (2011). Generation of reactive oxygen species in 1-methyl-4-phenylpyridinium (MPP+) treated dopaminergic neurons occurs as an NADPH oxidase-dependent two-wave cascade. J Neuroinflammation.

[57] Zeevalk GD, Razmpour R, Bernard LP (2008). Glutathione and Parkinson’s disease: is this the elephant in the room?. Biomed Pharmacother.

[58] Zimmer L, Delpal S, Guilloteau D, Aïoun J, Durand G, Chalon S (2000). Chronic n-3 polyunsaturated fatty acid deficiency alters dopamine vesicle density in the rat frontal cortex. Neurosci Lett.

